# *Streptomyces*-linked exercise gene networks orchestrate immune exclusion and determine immunotherapy response in hepatocellular carcinoma

**DOI:** 10.1371/journal.pone.0356502

**Published:** 2026-08-20

**Authors:** Xiaoting Zhao, Yu He, Yibin Zhang, Dapeng Liu, Yingwei Wang, Zuopeng Wang, Yudiao Huang

**Affiliations:** 1 TCM Teaching and Research Department, College of Pharmacy, Daqing Campus, Harbin Medical University, Daqing, Heilongjiang Province, P.R.China; 2 Department of Oncology, Daqing Oilfield General Hospital, Daqing, Heilongjiang Province, P.R.China; 3 Department of Physical Education, Daqing Normal University, Daqing, Heilongjiang Province, P.R.China; 4 Department of Physical Education and Teaching Research, Shantou University, Shantou, Guangdong Province, P.R.China; 5 Department of Neurology, The Fifth Affiliated Hospital of Harbin Medical University, Daqing, China; Xiangya Hospital Central South University, CHINA

## Abstract

Exercise-related genes (ERGs) have emerged as potential modulators of tumor biology, yet their systematic characterization in hepatocellular carcinoma (HCC) remains incomplete. Here we integrated 320 ERGs from MSigDB with TCGA-HCC cohort to construct an interaction-perturbation network, identifying two distinct subtypes with divergent prognostic outcomes. Cluster1 (44.4% of patients) exhibited significantly higher network perturbation scores, activated proliferation and epithelial-mesenchymal transition pathways, and elevated immune checkpoint gene expression, collectively contributing to poorer survival. This subtype also demonstrated enhanced immune evasion potential and lower predicted immunotherapy response rates. Conversely, Cluster2 (55.6%) was characterized by metabolic pathway enrichment, increased *CTNNB1* mutations, higher tumor mutational burden, and more favorable immunotherapy prediction. Notably, integrated analysis of intratumoral microbiota revealed that *Streptomyces* abundance was significantly associated with immune exclusion features and negatively correlated with both exercise-related and lactate metabolic pathways. Mediation analysis further suggested that elevated lactate metabolism was statistically associated with adaptive microbial changes, which in turn showed directional associations with exercise-related pathway activity and clinical outcomes. Using *Streptomyces*-associated host genes, we developed an 8-gene prognostic signature that effectively stratified patient outcomes and predicted immunotherapy response across multiple independent cohorts. Our findings delineate exercise-related molecular subtypes with distinct immune-microbiota-metabolic crosstalk and provide a clinically applicable signature for risk stratification in HCC.

## Introduction

Hepatocellular carcinoma (HCC) persists as a major cause of cancer-related deaths on a global scale, with rising incidence and limited therapeutic options for advanced disease [1]. Although immune checkpoint inhibitors (ICIs) have revolutionized the treatment landscape for HCC [2,3], clinical responses vary considerably among patients, underscoring an urgent need to better understand the determinants of immunotherapy efficacy and to develop reliable biomarkers for patient stratification [4]. Recent evidence has highlighted the intricate crosstalk between tumor cell-intrinsic programs, the immune microenvironment, and commensal microorganisms in shaping cancer progression and treatment outcomes [5–8], yet the molecular underpinnings of these interactions remain largely unexplored in HCC.

Exercise-related genes (ERGs) have recently garnered attention for their potential roles in modulating tumor biology beyond their classical functions in muscle physiology [9–11]. Emerging studies suggest that ERG expression patterns may influence cancer cell proliferation, metabolic reprogramming, and immune surveillance [9,12]. However, systematic characterization of ERGs in HCC, particularly their integration with tumor microenvironment features and microbial communities, has not been performed. Meanwhile, accumulating evidence implicates the intratumoral microbiota as an active participant in cancer pathogenesis, capable of modulating local immune responses and even influencing response to immunotherapy [13–15]. Specific microbial genera, including *Streptomyces*, have been detected in various tumor types [16–18], but their functional significance in HCC remains poorly understood.

Notably, the interplay among exercise-related gene networks, tumor metabolism, and microbial composition represents an unexplored dimension of HCC biology. Lactate metabolism, a key pathway linking cellular energetics to immune function, may serve as a critical node connecting these elements [19–21]. Whether lactate-driven metabolic alterations shape the intratumoral microbiota, and whether microbial changes in turn modulate exercise-related pathways and immune evasion, has not been investigated. Furthermore, the clinical implications of such multi-directional interactions for patient prognosis and immunotherapy response warrant systematic evaluation.

Here we integrated exercise-related gene signatures with transcriptomic, genomic, and clinical data from HCC cohorts, coupled with intratumoral microbiota profiling and causal mediation analysis, to delineate molecular subtypes with distinct tumor microenvironment features and therapeutic vulnerabilities. We identified *Streptomyces* as a key microbial genus associated with immune exclusion, and uncovered directional relationships linking lactate metabolism, microbial adaptation, and exercise-related pathway activity. Leveraging these insights, we developed and validated a prognostic gene signature with potential utility for predicting immunotherapy response, providing a framework for personalized treatment strategies in HCC.

## Results

### Identification of two distinct subtypes based on exercise-related genes

**Fig 1:**
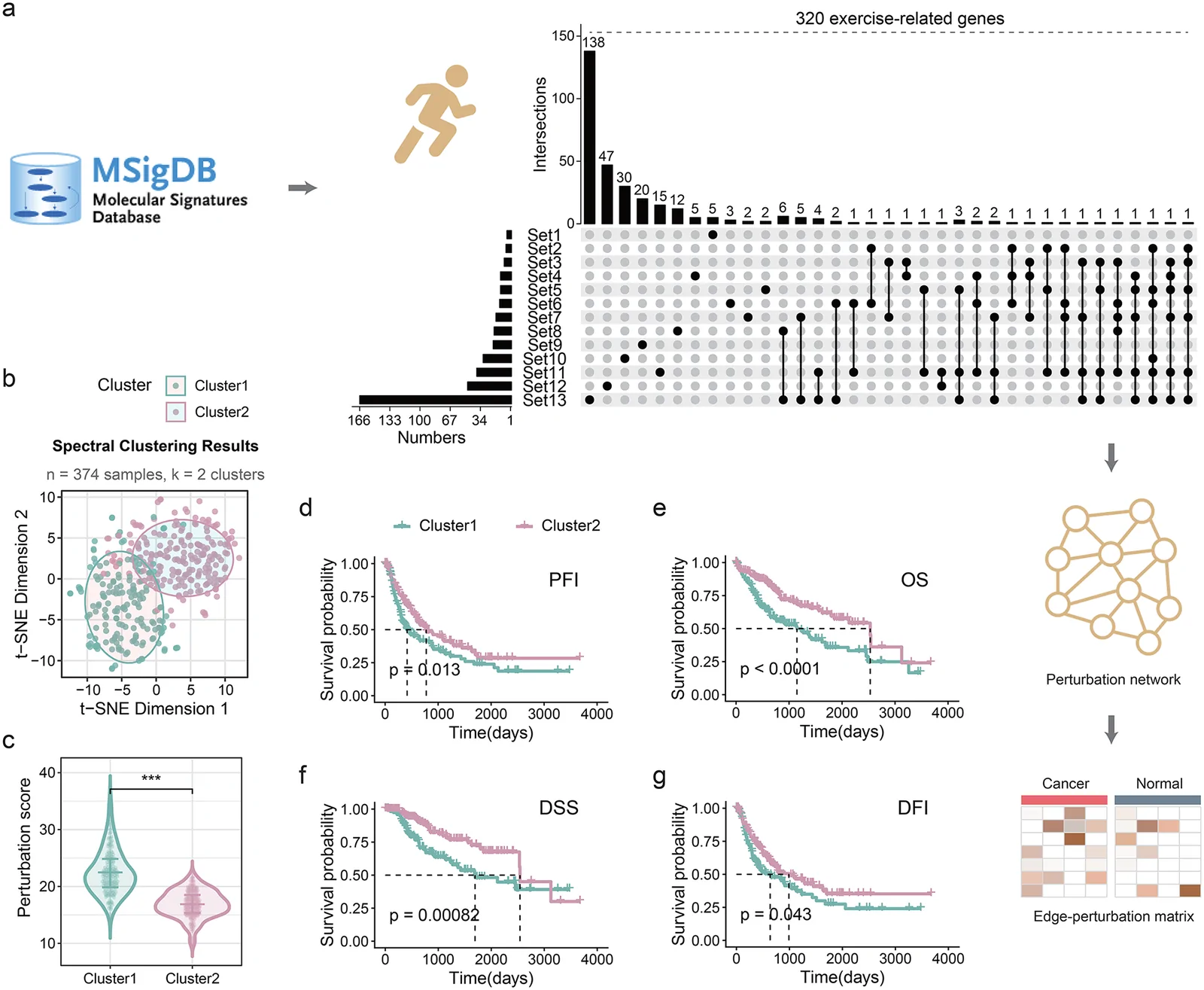
Identification of two distinct subtypes based on exercise-related gene interaction networks. (a) Overlap of exercise-related genes (ERGs) from 13 MSigDB gene sets, yielding 320 unique ERGs. (b) Consensus clustering of 374 TCGA-HCC samples based on the interaction perturbation matrix, showing two clusters (Cluster1, n = 166; Cluster2, n = 208). (c) Comparison of network perturbation scores between Cluster1 and Cluster2 (Wilcoxon rank-sum test, *P* < 0.001). (d-g) Kaplan-Meier survival curves comparing (d) progression-free interval (PFI), (e) overall survival (OS), (f) disease-specific survival (DSS), and (g) disease-free interval (DFI) between the two subtypes. *P*-values were calculated using the log-rank test. ***P < 0.001.

Initially, thirteen exercise-related gene sets were retrieved from the MSigDB database (Supplementary Table 1). The pathway activity of these gene sets exhibited strong correlations with features of the tumor microenvironment (Fig. S1 in S1 File). The size of individual gene sets varied from 5 to 166 genes (Fig 1a). Among them, the HP_EXERCISE_INTOLERANCE set contained 166 genes, while the WP_EXERCISEINDUCED_CIRCADIAN_REGULATION set comprised 48 genes. Collectively, these thirteen gene sets encompassed 320 non-redundant exercise-related genes (ERGs) (Supplementary Table 2 in S2 File). Mapping these 320 genes onto the TCGA-HCC cohort yielded 280 ERGs. A Pearson correlation analysis was performed between the expression levels of these 280 genes and the immune scores of the samples, resulting in the identification of 162 ERGs that were significantly associated with immunity (Fig. S2 in S1 File). To construct an interaction-perturbation matrix for these ERGs, we applied a previously established network-based pipeline [22]. A background network was first defined, consisting of 155 nodes (Supplementary Table 3) and 5,201 edges (Supplementary Table 4), where an edge represented an absolute correlation coefficient greater than 0.3 and an adjusted P-value less than 0.05 between two ERGs. Subsequently, an expression rank matrix was generated by ranking the expression of each gene within each sample. This matrix was then transformed into a delta rank matrix based on interactions from the background network. Given that gene interactions in normal tissues are known to be more stable and conservative than those in tumor tissues [22], we used the average expression vector from all normal samples as a benchmark. For each tumor sample, a delta rank vector was calculated relative to this benchmark, generating an interaction perturbation matrix that captures the sample-specific perturbation of ERG interactions.

Focusing on the most variable and differentially regulated interactions, we identified 1,791 edges (Supplementary Table 5) common to the top 3,000 most differentially expressed edges between tumor and adjacent normal tissues and the top 3,000 edges with the highest variance across tumor samples. These edges involved 129 genes. Utilizing the interaction-perturbation matrix, we performed consensus clustering on the 374 HCC samples from the TCGA cohort, which delineated two distinct subtypes: Cluster1 (166 patients, 44.4%) and Cluster2 (208 patients, 55.6%) (Fig 1b). Notably, the network perturbation score was significantly elevated in Cluster1 compared to Cluster2 (Fig 1c, P < 0.001). Survival analyses revealed that this heightened network instability in Cluster1 was associated with markedly poorer clinical outcomes, as evidenced by shorter progression-free interval (PFI), overall survival (OS), disease-specific survival (DSS), and disease-free interval (DFI) (Fig 1d-1g).

### Distinct tumor microenvironment and immune evasion characteristics between subtypes

**Fig 2:**
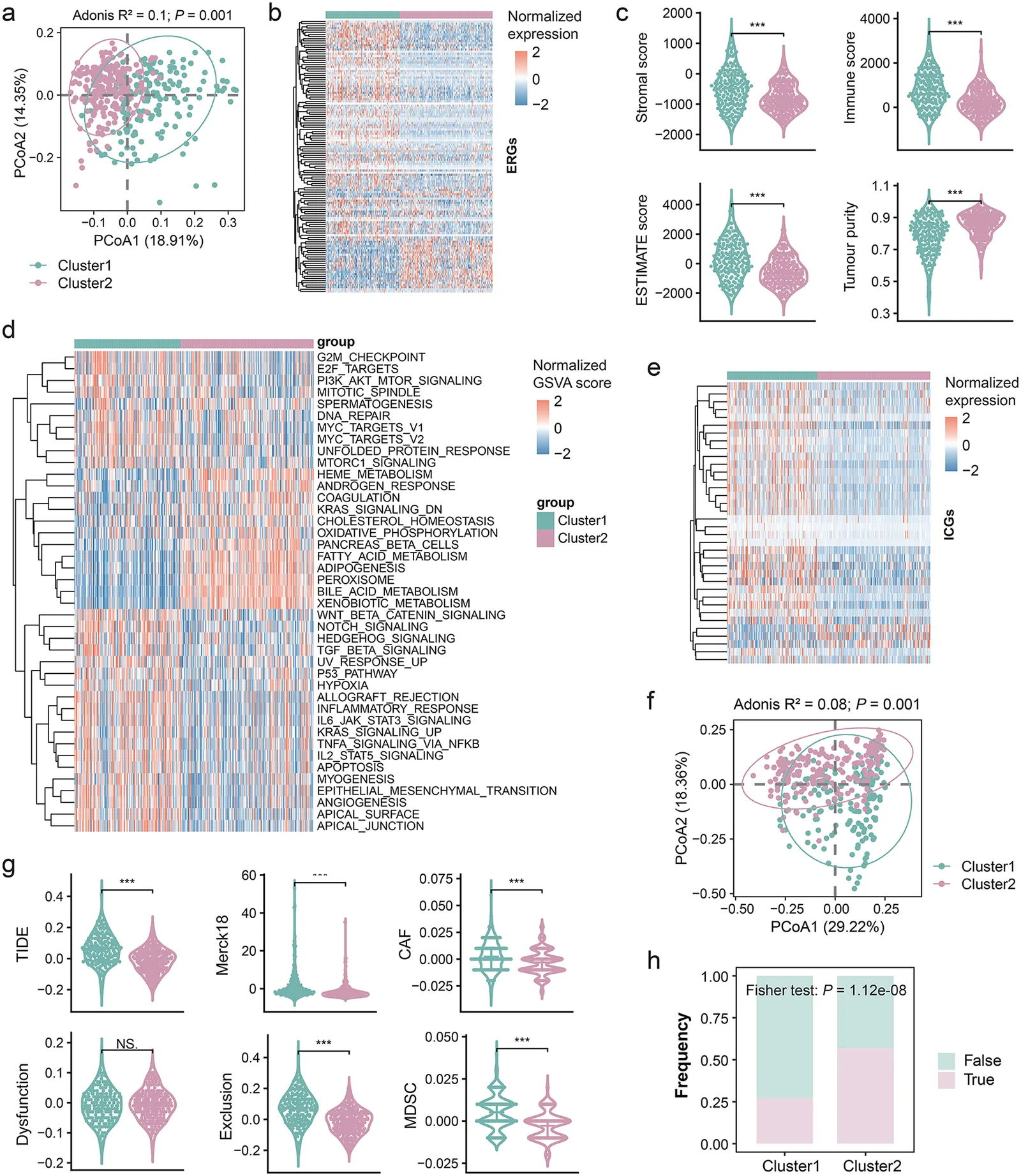
Distinct tumor microenvironment and immune evasion characteristics between subtypes. (a-b) Different expression patterns of ERGs between Cluster1 and Cluster2. (c) Comparison of stromal scores, immune scores, ESTIMATE scores, and tumor purity between subtypes (Wilcoxon rank-sum test). (d) Heatmap showing the GSVA enrichment scores of hallmark pathways in Cluster1 and Cluster2. (e-f) Different expression profiles of immune checkpoint genes (ICGs) between subtypes. (f) Proportion of ICGs showing significant upregulation in each subtype. (g) Comparison of immunotherapy-related scores, including TIDE, Merck18, CAF, dysfunction, exclusion and MDSC scores, between subtypes (Wilcoxon rank-sum test). (h) Proportion of potential immunotherapy responders (TIDE score < 0) and non-responders (TIDE score ≥ 0) in each subtype (Fisher’s exact test, P = 1.1e-08). ***P < 0.001; **P < 0.01; *P < 0.05; ns, not significant.

The two subtypes exhibited significant differences in ERG expression patterns (Fig 2a, P = 0.001), with a greater number of ERGs being upregulated in Cluster1 (Fig 2b). GSVA scores were computed for the thirteen exercise-related gene sets, and eleven of these sets exhibited significant differences between the two subtypes (Fig. S3 in S1 File). Cluster1 displayed significantly higher stromal, immune, and ESTIMATE scores, coupled with lower tumor purity, relative to Cluster2 (Fig 2c). To explore functional pathway heterogeneity, we performed GSVA enrichment analysis (Fig 2d). Cluster1 was characterized by activation of cell proliferation-related pathways, including G2M checkpoint, MYC targets V1, and MYC targets V2. Conversely, Cluster2 showed enrichment of various metabolic pathways, such as bile acid metabolism and fatty acid metabolism. Additionally, Cluster1 exhibited activation of epithelial-mesenchymal transition and angiogenesis pathways, which may contribute to the poorer prognosis observed in this subtype. The expression profiles of immune checkpoint genes (ICGs) also diverged significantly between the groups, with a higher proportion being upregulated in Cluster1 (Fig 2e and 2f).

Given the pivotal role of the tumor immune microenvironment in determining immunotherapy response in HCC, we evaluated potential differences in immune evasion mechanisms. The Tumor Immune Dysfunction and Exclusion (TIDE) score, a predictor of immunotherapy response, was significantly higher in Cluster1 (P < 0.001), suggesting a higher likelihood of poor response (Fig 2g). Furthermore, scores for Merck18, cancer-associated fibroblasts (CAF), exclusion, and myeloid-derived suppressor cells (MDSC) were all significantly elevated in Cluster1, while dysfunction scores were comparable between the subtypes. Stratifying patients based on TIDE score (dichotomized at zero) into potential responders and non-responders revealed that the proportion of potential responders was substantially lower in Cluster1 (45/166, 27.1%) than in Cluster2 (118/208, 56.7%) (Fig 2h, *P* = 1.1e-08), underscoring the differential immunotherapy efficacy predicted for each subtype.

### Clinicopathological and genomic landscapes of the two subtypes

**Fig 3:**
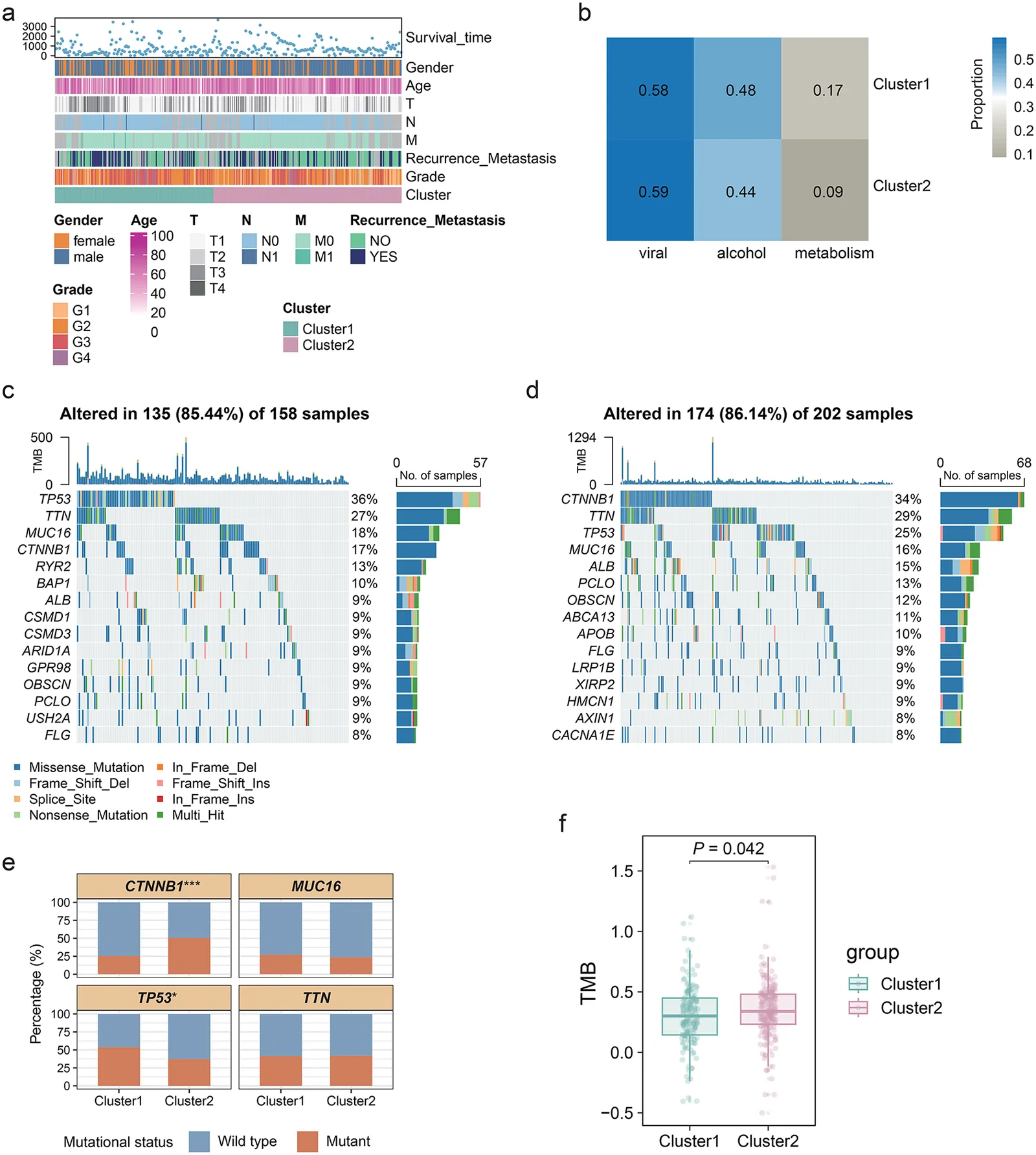
Clinicopathological and genomic landscapes of the two subtypes. (a) Distribution of clinical features, including age, sex, and tumor stage, across Cluster1 and Cluster2. (b) Etiology composition of HCC cases in each subtype (viral, alcohol-related, metabolic dysfunction-associated). (c,d) Oncoplots displaying the most frequently mutated genes in (c) Cluster1 and (d) Cluster2. (e) Comparison of mutation frequencies for CTNNB1 and TP53 between subtypes (Fisher’s exact test). (f) Tumor mutational burden (TMB) comparison between Cluster1 and Cluster2 (Wilcoxon rank-sum test, *P* = 0.042).

Comparative analysis of clinical features revealed that patients in Cluster2 were generally older and had a higher proportion of advanced-stage disease compared to those in Cluster1 (Fig 3a). Both subtypes were predominantly composed of virus-related and alcohol-related HCC cases (Fig 3b). Notably, the proportion of metabolic dysfunction-associated HCC was considerably lower in Cluster2 than in Cluster1. At the genomic level, TP53, TTN, MUC16, and CTNNB1 were the most frequently mutated genes in both subtypes (Fig 3c and 3d). However, their mutation frequencies differed significantly: CTNNB1 mutations were significantly more prevalent in Cluster2 (Fig 3e, *P* < 0.001), whereas TP53 mutations were enriched in Cluster1 (Fig 3e, *P* = 0.011). Additionally, tumor mutational burden (TMB) was significantly higher in Cluster2 (Fig 3f, *P* = 0.042), aligning with the predicted better immunotherapy response for this subtype.

### Divergent lactate metabolism and intratumoral microbiota composition

**Fig 4:**
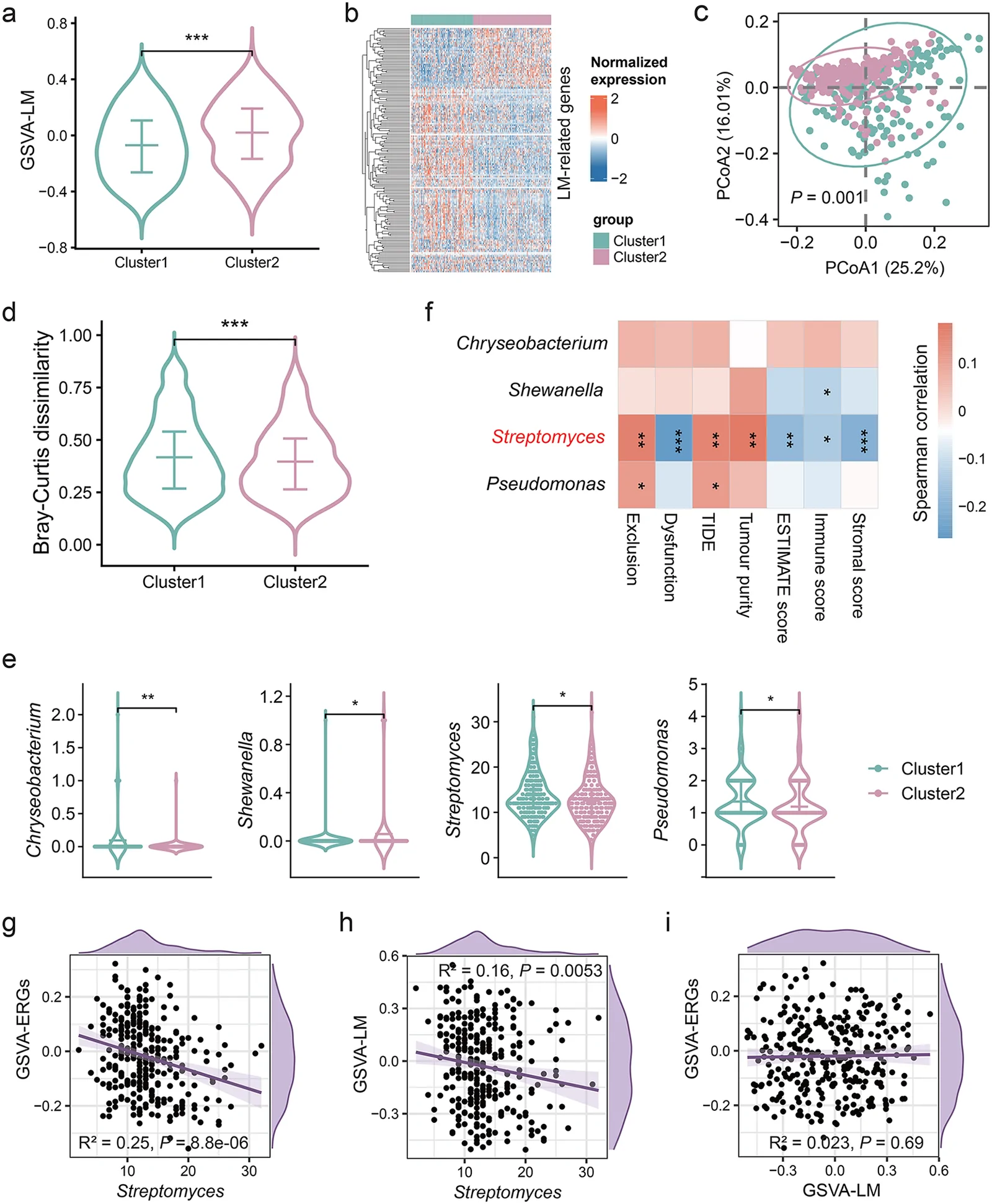
Divergent lactate metabolism and intratumoral microbiota composition between subtypes. (a) GSVA scores for the lactate metabolism pathway in Cluster1 and Cluster2 (Wilcoxon rank-sum test, P < 0.001). (b) Heatmap showing expression patterns of lactate metabolism-related genes across subtypes. (c) Overall comparison of lactate metabolism gene expression between subtypes (gene set enrichment analysis, P = 0.001). (d) Beta diversity analysis using Bray-Curtis dissimilarity, showing significant separation between subtypes (PERMANOVA, P < 0.001). (e) Box plots illustrating relative abundances of four differentially abundant genera (Chryseobacterium, Shewanella, Streptomyces, Pseudomonas) between subtypes (DESeq2, adjusted P < 0.05). (f) Heatmap of Spearman correlations between these four genera and tumor microenvironment features (immune score, stromal score, TIDE components, tumor purity). (g) Correlation between Streptomyces abundance and exercise-related pathway activity (Spearman correlation, P = 8.8e-06). (h) Correlation between Streptomyces abundance and lactate metabolism pathway activity (Spearman correlation, P = 0.0053). (i) Correlation between exercise-related pathway activity and lactate metabolism pathway activity (Spearman correlation, P = 0.69). LM, lactate metabolism.

We next investigated differences in lactate metabolism, a key pathway linking metabolism and immunity. The GSVA score for the lactate metabolism pathway was significantly higher in Cluster2 than in Cluster1 (Fig 4a, *P* < 0.001), and the expression patterns of lactate metabolism-related genes were distinctly different between the two groups (Fig 4b and 4c, *P* = 0.001). Examination of the tumor microbial community structure revealed no significant difference in alpha diversity between the subtypes (Fig. S4 in S1 File). However, beta diversity, a measure of community dissimilarity, was significantly greater in Cluster1 (Fig 4d, *P* < 0.001), indicating a more heterogeneous microbial composition. Differential abundance analysis identified four genera, including *Chryseobacterium*, *Shewanella*, *Streptomyces*, and *Pseudomonas*, with significantly different relative abundances between the subtypes (Fig 4e). Correlation analysis of these genera with tumor microenvironment features (Fig 4f) showed that Streptomyces abundance was positively correlated with exclusion score, TIDE, and tumor purity, while being negatively correlated with dysfunction score, stromal score, and immune score. Furthermore, *Streptomyces* abundance exhibited significant negative correlations with both exercise-related pathway activity (Fig 4g, *P* = 8.8e-06) and lactate metabolism pathway activity (Fig 4h, *P* = 0.0053). Interestingly, no significant correlation was observed between the activities of the exercise-related and lactate metabolism pathways themselves (Fig 4i, *P* = 0.69).

### Causal relationships among *Streptomyces*, lactate metabolism, and exercise-related pathways

**Fig 5:**
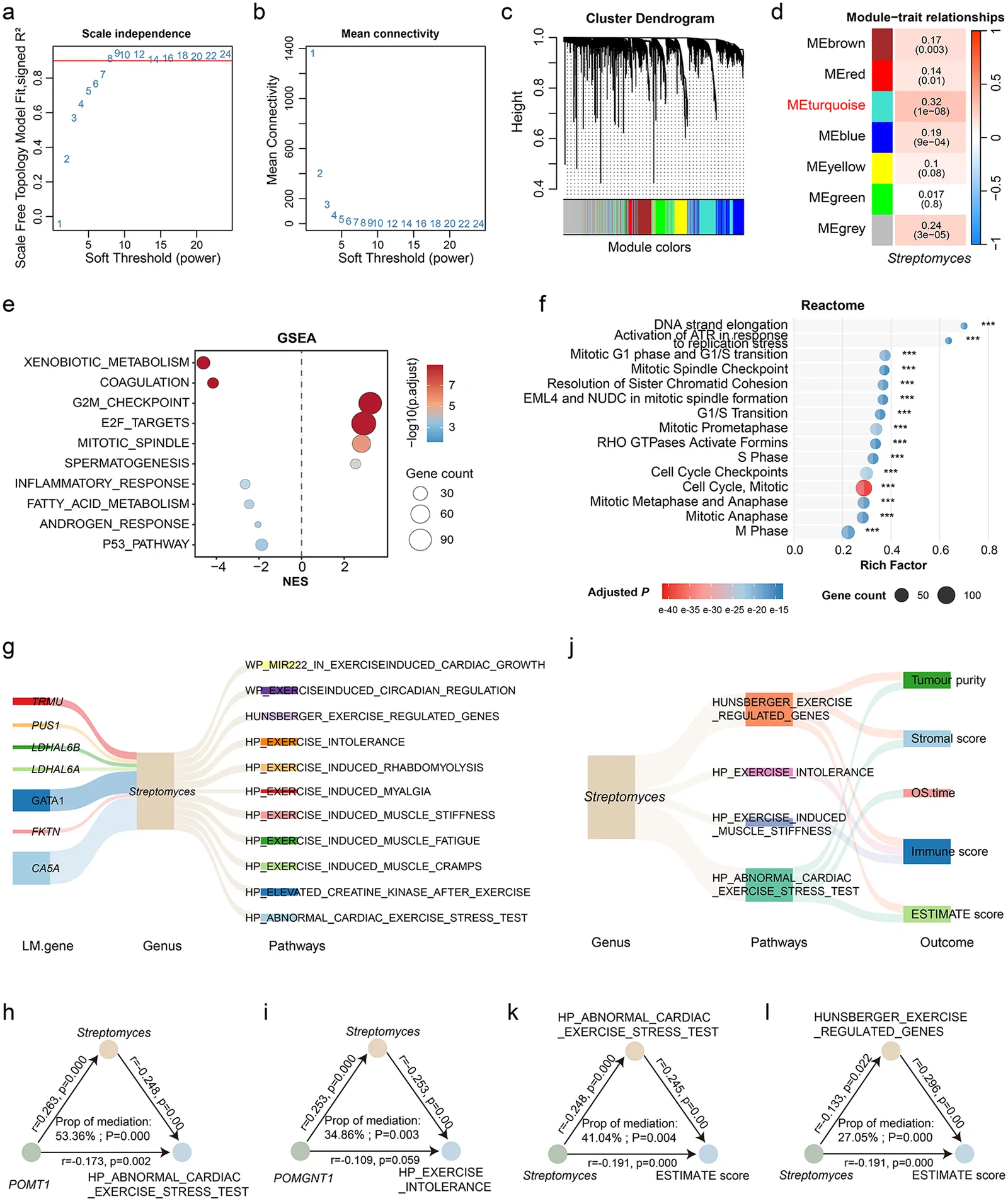
Causal mediation analysis reveals directional relationships among Streptomyces, lactate metabolism, and exercise-related pathways. (a-b) Scale-free fit index and mean connectivity analysis for selecting the soft-thresholding power in WGCNA. (c) Cluster dendrogram showing co-expression modules identified by WGCNA. (d) Heatmap of module-trait relationships, displaying correlations between module eigengenes and Streptomyces abundance. The turquoise module showed the strongest association. Functional enrichment results of genes in turquoise module by (e) GSEA and (f) Reactome. (g) Causal mediation model with individual LM-related gene expression levels as independent variables, *Streptomyces* abundance as the mediator, and exercise-related pathway activity (GSVA score) as the dependent variable. Significant mediation paths were identified. (h-i) Representative mediation links among individual LM-related gene expression, *Streptomyces* abundances, and exercise-related pathway activity (GSVA score). (j) Mediation analysis with *Streptomyces* abundance as the independent variable, exercise-related pathway activity (GSVA score) as the mediator, and TME features or survival outcomes as dependent variables. (k-l) Representative mediation links among *Streptomyces*, exercise-related pathway and ESTIMATE score.

Next, we sought to investigate the biological functions of host genes driven by the genus *Streptomyces*. Using weighted gene co-expression network analysis (WGCNA), we partitioned the genes into modules and identified the module most significantly correlated with the relative abundance of *Streptomyces*, containing 1,602 genes (Fig 5a-5d). Gene ontology (GO) functional enrichment analysis of the genes in this module revealed enrichment in numerous pathways related to energy, motility, and immunity (Fig. S5 in S1 File). Gene set enrichment analysis (GSEA) and Reactome enrichment analyses indicated that the module genes were primarily enriched in cell proliferation-related pathways, such as G2M_CHECKPOINT, E2F_TARGETS, Mitotic G1 phase and G1/S transition, and Cell Cycle Checkpoints (Fig 5e and 5f).

To explore statistically directional relationships between *Streptomyces* abundance, lactate metabolism, and exercise-related pathways, we performed mediation analysis. When individual lactate metabolism-related gene expression levels were considered as the independent variable and exercise-related pathway activity (GSVA score) as the dependent variable, numerous significant mediation paths involving *Streptomyces* abundance as the mediator were identified (Fig 5g; Supplementary Table 6). For example, *POMT1* influenced the activity of the HP_ABNORMAL_CARDIAC_EXERCISE_STRESS_TEST pathway through its effect on *Streptomyces* abundance (Fig 5h). Additionally, the genus *Streptomyces* mediated the relationship between *POMGNT1* expression and the HP_EXERCISE_INTOLERANCE pathway (Fig 5i). In contrast, when individual ERG expression levels were set as independent variables and lactate metabolism pathway activity (GSVA score) as the dependent variable, no significant mediation effects were detected (Supplementary Table 7). This pattern suggests that elevated lactate metabolism in the liver tumor microenvironment may associate with adaptive changes in the microbiota, and the latter shows directional associations with the activity of exercise-related pathways.

Next, given the strong associations of exercise-related pathways with both the tumor microenvironment and prognosis, we again conducted causal mediation analysis to examine the relationships among the genus *Streptomyces*, exercise pathways, and either microenvironmental features or prognostic outcomes. Numerous significant pathways were detected (Fig 5j; Supplementary Table 8). These pathways spanned cascades from *Streptomyces* to exercise pathways, and subsequently to key endpoints such as tumor immunity and overall survival. For example, our analysis revealed statistically significant indirect effects of Streptomyces on microenvironmental features through the HP_ABNORMAL_CARDIAC_EXERCISE_STRESS_TEST and HUNSBERGER_EXERCISE_REGULATED_GENES pathways (Fig 5k and 5l).

### Development of a Streptomyces-driven prognostic gene signature

**Fig 6:**
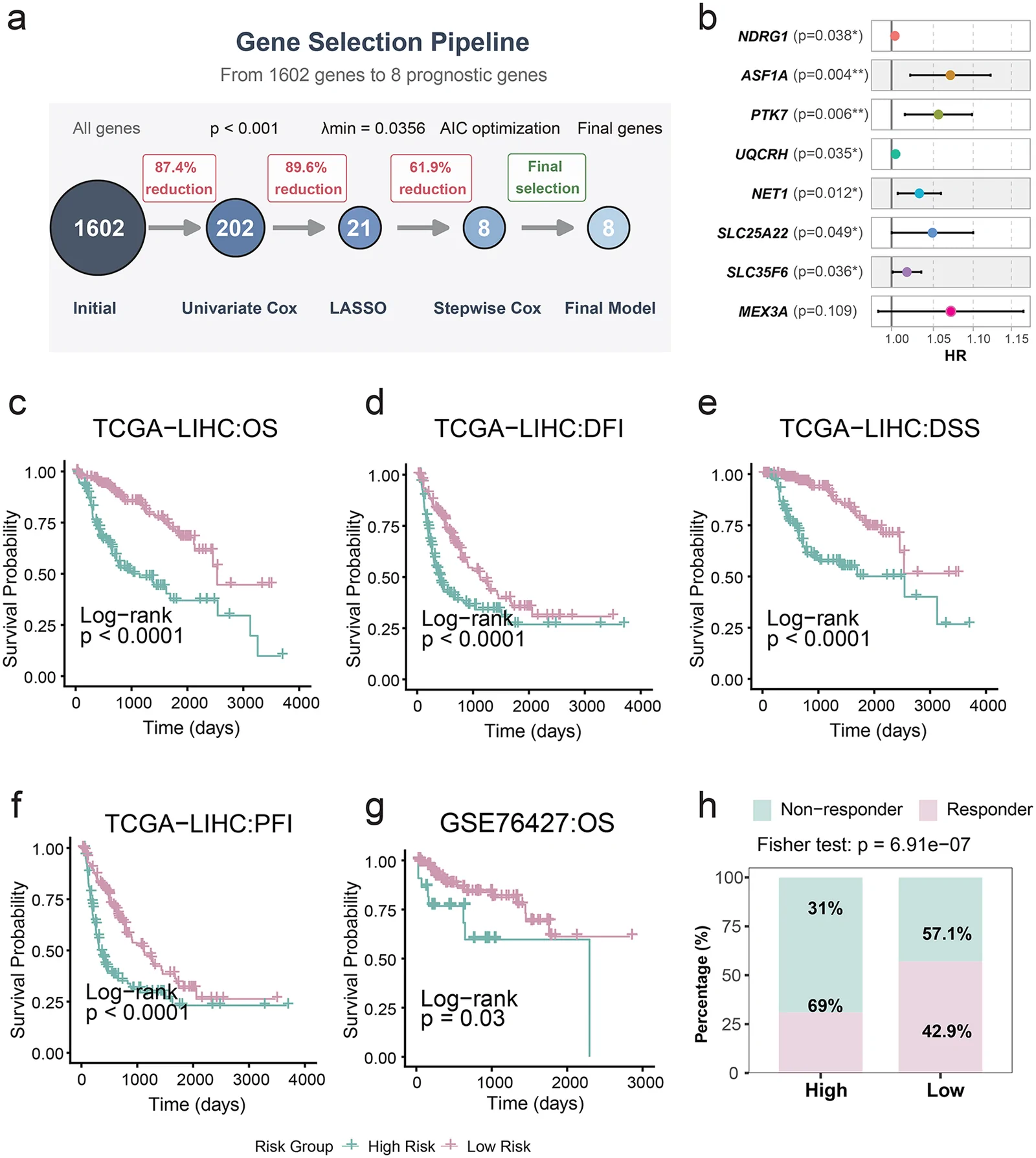
Development and validation of an 8-gene Streptomyces-driven prognostic signature. (a) Pipeline of key gene selection. (b) Forest plot showing the association between eight genes and OS. (c-f) Kaplan-Meier survival curves for high-risk and low-risk groups stratified by the 8-gene signature in the TCGA cohort (log-rank test). (g) Validation of prognostic performance in independent GEO cohort (GSE76427). (h) Immunotherapy response prediction in the GSE109211 cohort, showing proportion of responders and non-responders in high-risk and low-risk groups (Fisher’s exact test).

Next, we sought to construct a prognostic model for HCC using host genes driven by the genus *Streptomyces*. Through univariate Cox regression, Lasso regression analysis, and stepwise Cox regression, eight key genes were ultimately selected (Fig 6a and 6b), and their coefficients were calculated (Supplementary Table 9). Subsequently, a formula was derived to compute the risk score for each patient: Risk score = NDRG1 × 0.003921 + ASF1A × 0.068658 + PTK7 × 0.054736 + UQCRH × 0.004658 + NET1 × 0.032415 + SLC25A22 × 0.047724 + SLC35F6 × 0.017933 + MEX3A × 0.069181

Patients were divided into high- and low-risk groups based on the median risk score. In the TCGA-LIHC cohort, significant differences in prognosis were observed between the two groups for OS, DFI, DSS, and PFI (Fig 6c-6f). Moreover, the performance of the prognostic model was validated in an independent dataset, GSE76427 (Fig 6g). We also introduced an HCC immunotherapy cohort, GSE109211, which included 21 responders and 46 non-responders. After stratifying patients into high- and low-risk groups, we found that patients in the high-risk group exhibited poorer responses to immunotherapy compared with those in the low-risk group (Fig 6h). To further test the independent prognostic ability of the model, we included the risk score and various clinical variables in a multivariate Cox regression model. The results showed that the risk score remained significantly associated with patient OS, thereby demonstrating its independent prognostic capability (Fig. S6 in S1 File).

## Discussion

In this study, we systematically characterized exercise-related gene (ERG) networks in HCC and uncovered their intricate interplay with tumor metabolism, immune microenvironment, and microbial composition. By integrating multi-omics data with causal mediation analysis, we identified two distinct molecular subtypes with divergent prognostic and immunotherapeutic trajectories, and demonstrated that *Streptomyces* abundance serves as a critical link connecting lactate metabolism to exercise-related pathway activity and clinical outcomes. These findings advance our understanding of the complex ecosystem within HCC and provide a clinically actionable framework for patient stratification.

The identification of two ERG-derived subtypes with opposing tumor microenvironment phenotypes aligns with the emerging paradigm that ERGs participate in cancer biology beyond their canonical roles [11]. Cluster1, characterized by high network perturbation, activated proliferation and EMT pathways, and elevated immune checkpoint expression, resembles an “immune-inflamed” yet immunosuppressed phenotype that paradoxically exhibits poor prognosis and immunotherapy resistance. This seemingly contradictory phenomenon can be explained by the fact that the quantity of immune infiltration does not necessarily equate to the quality of immunity. In Cluster 1, the simultaneous activation of EMT and angiogenesis pathways, the increase in TIDE/immune exclusion score, the increase in MDSC and CAF infiltration, and the upregulation of ICGs, collectively create an immunosuppressive and immune-excluded microenvironment. Additionally, the lower TMB in Cluster 1 may limit the accessibility of new antigens and the recognition by T cells. Therefore, this “immune inflammation but immunosuppression” phenotype reflects ineffective immune activation rather than protective anti-tumor immunity.

Conversely, Cluster2 displayed metabolic pathway enrichment, higher CTNNB1 mutation frequency, and increased TMB, features collectively associated with more favorable immunotherapy prediction [23–25]. The reciprocal relationship between CTNNB1 mutations (enriched in Cluster2) and TP53 mutations (enriched in Cluster1) recapitulates the well-established genomic dichotomy in HCC and extends its relevance to ERG networks and immunotherapy responsiveness. Notably, the higher TMB in Cluster2 provides a plausible mechanistic basis for the predicted better immunotherapy response, as neoantigen burden has been associated with checkpoint inhibitor efficacy across multiple cancer types [26,27]. These findings suggest that ERG-based subtyping captures clinically meaningful heterogeneity with direct therapeutic implications.

The most provocative finding of our study is the identification of *Streptomyces* as a key microbial genus associated with immune exclusion, exercise-related pathway suppression. While tumor-associated microbiota has emerged as an important modulator of cancer progression and treatment response [28,29], the specific role of *Streptomyces* in HCC has not been previously described. *Streptomyces* species are well-known for their prolific production of bioactive secondary metabolites, including numerous clinically used antibiotics and anticancer agents [30,31]. A recently study found that intratumoral microbiota differs among HCC subtypes stratified by lactate metabolism, with *Streptomyces* significantly enriched in the subtype with the worst prognosis [17]. Our observation that *Streptomyces* abundance correlates negatively with both exercise-related and lactate metabolism pathways raises intriguing questions about potential microbial-host metabolic crosstalk. It is tempting to speculate that *Streptomyces*-derived metabolites may directly modulate host gene expression programs or indirectly influence the tumor microenvironment through effects on immune cell function.

The mediation analysis provided statistical support for directional relationships among these components. Lactate metabolism is correlated with the abundance of *Streptomyces*, and the abundance of Streptomyces is associated with the exercise-related pathways. This finding suggests a model consistent with the impact of metabolic reprogramming on the composition of microorganisms. This directionality is supported by the absence of significant mediation effects when the order was reversed, strengthening the causal inference. The subsequent demonstration that exercise-related pathways mediate the effects of *Streptomyces* on immune features and survival further refines this model, positioning ERG networks as critical transducers of microbial influences on clinical outcomes.

The prognostic signature derived from *Streptomyces*-associated host genes demonstrated robust performance across multiple independent cohorts and extended its utility to predicting immunotherapy response in an external validation set. This 8-gene panel captures diverse biological processes including energy metabolism, cytoskeletal organization, and immune regulation, reflecting the multifaceted influence of microbial-host interactions on tumor behavior. The inclusion of genes involved in these pathways may render the signature more resilient to platform-specific variations and cohort effects, contributing to its generalizability. From a translational perspective, this signature could facilitate risk stratification and guide treatment decisions, particularly in identifying patients unlikely to benefit from immune checkpoint blockade who might be candidates for alternative or combinatorial approaches.

Several limitations of this study warrant consideration. First, the intratumoral microbiota data were derived from bulk RNA-seq using computational deconvolution rather than direct metagenomic sequencing, which may introduce bias and precludes species-level resolution. Validation studies using 16S rRNA or shotgun metagenomic sequencing in independent cohorts are needed to confirm the *Streptomyces* findings and explore potential functional contributions of specific microbial strains. Second, although our mediation analysis is statistically rigorous, it relies on cross-sectional data and model assumptions. Therefore, the directionality relationships inferred should be regarded as hypothetical propositions. In the future, experimental studies (such as co-culture systems or germ-free animal models) are needed to establish the true biological causal relationships. Third, the prognostic signature, although validated in multiple cohorts, was developed using retrospective data and requires prospective evaluation to establish clinical utility. Fourth, when constructing the interaction perturbation matrix, we assumed that the gene co-expression in the adjacent tissues was relatively stable and used it as the reference. However, the adjacent tissues of HCC often have liver cirrhosis or chronic inflammation, which may interfere with the co-expression pattern and thereby introduce bias to the perturbation score. Finally, due to the lack of physical activity and BMI data in TCGA, we were unable to directly correlate the ERG features with the actual physical activity status of the patients. Therefore, the “exercise-related” referred to in this study refers to the established gene/pathway associations in other physiological contexts, rather than the behavioral phenotypes of the patients.

In conclusion, this study provides a comprehensive framework integrating ERG networks, tumor metabolism, immune microenvironment, and microbial composition in HCC. The identification of *Streptomyces* as a key microbial determinant linked to immune exclusion opens new avenues for therapeutic exploration, while the derived prognostic signature offers a practical tool for patient stratification. Our findings underscore the importance of considering multi-directional interactions among tumor cells, immune system, and microbiota in understanding cancer biology and optimizing treatment strategies. Future work should focus on validating these observations in prospective cohorts and exploring the therapeutic potential of modulating the intratumoral microbiota to enhance immunotherapy efficacy in HCC.

## Methods

### Data acquisition and processing

Transcriptomic data, corresponding clinical information, and somatic mutation profiles for liver hepatocellular carcinoma (LIHC) were obtained from The Cancer Genome Atlas (TCGA) database (https://portal.gdc.cancer.gov/). All data were accessed on 10 November 2025. Samples with incomplete survival information were excluded, yielding 374 tumor samples and 50 adjacent normal tissues for subsequent analysis. For external validation, an independent HCC cohort (GSE76427) was retrieved from the Gene Expression Omnibus (GEO) database (accessed on 16 January 2026). GSE76427 is an independent cohort with no sample overlap with TCGA-LIHC. An additional immunotherapy-treated cohort (GSE109211) comprising HCC patients receiving immune checkpoint blockade was included to evaluate the predictive value of our signature for immunotherapy response (accessed on 20 January 2026). All gene expression data were log2-transformed and normalized where appropriate. The authors did not have access to information that could identify individual participants during or after data collection, because all data were obtained from de-identified public repositories (TCGA and GEO).

### Exercise-related gene collection and network construction

Exercise-related gene sets were downloaded from the Molecular Signatures Database (MSigDB, version v2026.1.Hs, accessed on 3 February 2026) using the search term “exercise”. A total of thirteen gene sets were retrieved. All the returned gene sets were included without any manual screening. Redundant genes across the gene sets were merged and duplicates were removed to obtain a comprehensive and unique unique exercise-related genes (ERGs). These ERGs were mapped to the TCGA-HCC expression matrix, retaining genes with detectable expression across samples. Immune scores for each sample were calculated using the ESTIMATE algorithm, and Pearson correlation analysis was performed to identify ERGs significantly associated with immune infiltration (*P* < 0.05).

To construct the ERG interaction network, pairwise Pearson correlation coefficients were calculated for all ERGs across tumor samples. Edges with absolute correlation coefficient greater than 0.3 and adjusted P-value less than 0.05 were retained to form the background network. Based on previous methodological work [22], we generated an expression rank matrix by ranking gene expression within each sample, which was then transformed into a delta rank matrix using interactions from the background network. Given that gene interactions in normal tissues exhibit greater stability compared to tumor tissues, the average expression vector of all normal samples was used as the benchmark. For each tumor sample, the deviation from this benchmark was calculated to generate a sample-specific interaction perturbation matrix.

### Identification of differential and variable interactions

To focus on the most biologically relevant interactions, we identified edges differentially regulated between tumor and adjacent normal tissues using the limma package, selecting the top 3,000 edges with the most significant changes. Concurrently, we calculated the variance of each edge across all tumor samples and selected the top 3,000 edges with the highest variance. The intersection of these two sets yielded a refined interaction network comprising 1,791 edges involving 129 genes, which was used for subsequent clustering analysis.

### Consensus clustering and subtype characterization

Based on the interaction perturbation matrix of the refined network, consensus clustering was performed using the ConsensusClusterPlus package in R. The optimal number of clusters was determined by examining the consensus matrix, cumulative distribution function, and relative change in area under the cumulative distribution curve. Kaplan-Meier survival analysis with log-rank tests was used to evaluate prognostic differences between identified subtypes for overall survival (OS), progression-free interval (PFI), disease-specific survival (DSS), and disease-free interval (DFI). Network perturbation scores were calculated as the mean absolute perturbation across all edges within each sample and compared between subtypes using the Wilcoxon rank-sum test.

### Tumor microenvironment and functional pathway analysis

Stromal scores, immune scores, and ESTIMATE scores were calculated using the ESTIMATE algorithm. Tumor purity was inferred from the ESTIMATE score using this formula:


$$\begin{array}{c} \text{Tumor purity }=\text{ cos}(\text{0}.\text{6049872018 }+\;\text{0}.\text{0001467884 }\times \text{ ESTIMATE score}) \end{array}$$

Gene set variation analysis (GSVA) was performed using the GSVA package to evaluate enrichment of hallmark gene sets from MSigDB. Differences in pathway activity between subtypes were assessed using the limma package with empirical Bayes moderation. Expression patterns of immune checkpoint genes (ICGs) were compared, and the proportion of ICGs upregulated in each subtype was calculated.

Immunotherapy response was predicted using the Tumor Immune Dysfunction and Exclusion (TIDE) algorithm (http://tide.dfci.harvard.edu/), which integrates dysfunction and exclusion signatures to estimate potential response to immune checkpoint blockade. Additional immunotherapy-related scores, including Merck18 (referring to the 18 IFN-γ-related gene expression characteristics associated with the response to ICIs), cancer-associated fibroblast (CAF), exclusion, dysfunction, and myeloid-derived suppressor cell (MDSC) scores, were obtained from the TIDE output. Patients were stratified into potential responders and non-responders based on TIDE score (dichotomized at zero), and the proportion of responders was compared between subtypes using Fisher’s exact test.

### Genomic and clinical feature analysis

Clinical characteristics, including age, sex, tumor stage, and etiology (viral, alcohol-related, metabolic dysfunction-associated), were compared between subtypes using appropriate statistical tests. Somatic mutation data were analyzed using the maftools package. Mutation frequencies for individual genes were calculated and compared between subtypes using Fisher’s exact test. Tumor mutational burden (TMB) was defined as the total number of nonsynonymous mutations per megabase and compared using the Wilcoxon test.

### Lactate metabolism analysis

A lactate metabolism-related gene set was compiled from previous publications [32]. GSVA was used to calculate a lactate metabolism score for each sample, which was compared between subtypes. Expression patterns of individual lactate metabolism genes were visualized using heatmaps, and overall expression differences were assessed using the gene set variation analysis framework.

### Intratumoral microbiota profiling and analysis

The tumor-resident microbiome data for HCC used in this study were derived from a recently published pan-cancer resource by Sheng et al [33]. Briefly, the authors reanalyzed raw RNA sequencing data of 33 cancer types from TCGA using an improved microbial profiling pipeline designed to minimize host contamination. To ensure specificity, sequencing reads that did not align to the human reference genome (hg38) were extracted and taxonomically classified against the NCBI non-redundant and fungi databases using Kraken 2, with abundance estimation subsequently refined by Bracken. A rigorous decontamination protocol was applied to remove potential background contaminants. This involved batch-wise identification of contaminating features using the decontam R package based on sequencing plates, as well as the exclusion of genera typically found in negative reagent controls. Only microbial species that passed this decontamination step were retained for downstream analysis. For the present study, we directly obtained the species-level relative abundance matrix of tumor-resident bacteria for HCC samples from the publicly available database (TCMbio, https://microbiomex.sdu.edu.cn/), established by the authors. This provided the normalized microbial profiles for subsequent analyses.

Alpha diversity was assessed using the Shannon and Simpson indices and compared between subtypes using the Wilcoxon test. Beta diversity was evaluated using Bray-Curtis dissimilarity, and significance of separation between subtypes was tested using permutational multivariate analysis of variance (PERMANOVA) with 999 permutations. Differential abundance analysis at the genus level was performed using the Wilcoxon test. *P* values were adjusted for multiple comparisons using the Benjamini-Hochberg false discovery rate (FDR) method. Genera with adjusted *P* < 0.05 were considered significantly different.

Correlation between differentially abundant genera and tumor microenvironment features (immune scores, stromal scores, TIDE components) was assessed using Spearman correlation. Associations between *Streptomyces* abundance and pathway activities (exercise-related and lactate metabolism) were evaluated similarly. For all the correlation analyses, the Benjamini-Hochberg FDR method was used to correct for multiple comparisons of *P* values. Correlations with *P* < 0.05 after FDR correction were considered statistically significant.

### Weighted gene co-expression network analysis

To identify host genes associated with Streptomyces abundance, we performed weighted gene co-expression network analysis (WGCNA) using the WGCNA package. Genes with variance in the top 50% across samples were included. A signed adjacency matrix was constructed using a soft-thresholding power selected based on scale-free topology fit. Modules were identified using the dynamic tree cut method with a minimum module size of 50 genes. Module-trait relationships were calculated by correlating module eigengenes with *Streptomyces* abundance. The module showing the strongest correlation was selected for functional enrichment analysis using clusterProfiler, with Gene Ontology (GO) and Kyoto Encyclopedia of Genes and Genomes (KEGG) pathway enrichment assessed.

### Mediation analysis for directional associations

Causal mediation analysis was performed using the mediation package in R to dissect directional relationships among Streptomyces abundance, lactate metabolism, exercise-related pathway activity, and clinical outcomes. In each model, the independent variable (X), mediator (M), and dependent variable (Y) were specified, and the average causal mediation effect (ACME), average direct effect (ADE), and proportion mediated were estimated using nonparametric bootstrap with 1,000 resamples. Significance was determined based on 95% confidence intervals excluding zero. In all mediation models, “lactate metabolism-related gene expression” refers to the expression level of a single gene, while “lactate metabolism pathway activity” or “exercise-related pathway activity” refers to the GSVA score calculated for the corresponding gene set. Although this method can indicate the consistency of direction, it cannot prove the causal relationship; the results should be interpreted as the statistical mediation effect.

### Development and validation of prognostic signature

Genes from the *Streptomyces*-associated WGCNA module were subjected to further refinement using univariate Cox regression. Genes with *P* < 0.001 were retained, narrowing the pool to 202 genes. Then, least absolute shrinkage and selection operator (LASSO) was implemented in the glmnet package, with penalty parameter selected by 10-fold cross-validation. Finally, multivariate stepwise Cox regression further refined the selection to 8 genes and the risk score formula was derived: risk score = Σ(coef_i × exp_i), where coef_i represents the regression coefficient and exp_i represents the expression level of gene i (Supplementary Table 3). Patients were stratified into high-risk and low-risk groups based on the median risk score. Prognostic performance was evaluated using Kaplan-Meier survival analysis with log-rank test. The signature was validated in independent GEO cohort (GSE76427) using the same risk score formula and cutoff.

For immunotherapy response prediction, the risk score was calculated for patients in the GSE91061 melanoma cohort (as a surrogate immunotherapy-treated dataset). Response status (responder vs. non-responder) was compared between high-risk and low-risk groups using Fisher’s exact test.

### Statistical analysis

All statistical analyses were performed using R software (version 4.5.1). Continuous variables were compared using Wilcoxon rank-sum test (two groups) or Kruskal-Wallis test (multiple groups). Categorical variables were analyzed using chi-square test or Fisher’s exact test as appropriate. Survival differences were assessed using Kaplan-Meier curves with log-rank tests. Correlation analyses employed Spearman’s rank correlation coefficient unless otherwise specified. P-values were two-sided, with P < 0.05 considered statistically significant unless adjusted for multiple comparisons where indicated.

## Supporting information

S1 File Supplementary Figure 1–6. (DOCX)

S2 File Supplementary Table 1–9. (XLSX)
