# Supplementary material for: Streptomyces-linked exercise gene networks orchestrate immune exclusion and determine immunotherapy response in hepatocellular carcinoma
Source: PLoS One. 2026 Aug 20;21(8):e0356502. doi: 10.1371/journal.pone.0356502 (PMC13492822; doi:10.1371/journal.pone.0356502)
Supplement: Supplementary file 1 [file pone.0356502.s001.docx]

***Streptomyces*-linked exercise gene networks orchestrate immune exclusion and determine immunotherapy response in hepatocellular carcinoma**

Xiaoting Zhao^1#^, Yu He^2#^, Yibin Zhang^2^, Dapeng Liu^3^, Yingwei Wang^4*^, Zuopeng Wang^3*^, Yudiao Huang^5*^

^1^TCM Teaching and Research Department, College of Pharmacy, Daqing Campus, Harbin Medical University, Daqing 163319, Heilongjiang Province, P.R.China

^2^Department of Oncology, Daqing Oilfield General Hospital, Daqing 163001, Heilongjiang Province, P.R.China

^3^Department of Physical Education, Daqing Normal University, Daqing 163712, Heilongjiang Province, P.R.China

^4^Department of Physical Education and Teaching Research, Shantou University, Shantou 515063, Guangdong Province, P.R.China

^5^Department of Neurology, The Fifth Affiliated Hospital of Harbin Medical University, Daqing, 163319, China

#The authors contributed to this study equally

Correspondence: Yudiao Huang: 41655579@qq.com

Zuopeng Wang: 26287477@qq.com

Yingwei Wang: ywwang@stu.edu.cn

**Fig. S1. Heatmap showing the correlation between tumor microenvironment features and GSVA score of exercise-related gene sets**. The rows of the heatmap represent the gene sets, and the columns represent the tumor microenvironment characteristics. **P* < 0.05, ***P* < 0.01, ****P* < 0.001.

**Fig. S2. Venn diagram showing the number of ERGs significantly associated with immune score.** Among the 280 ERGs, 162 were significantly correlated with the immune score.

**Fig. S3. Violin plots showing the difference in GSVA score of exercise-related gene sets between Cluster1 and Cluster2.** **P* < 0.05, ***P* < 0.01, ****P* < 0.001. NS, not significant.

**Fig. S4. Violin plots showing the difference in alpha diversity of intratumoral microbiota between Cluster1 and Cluster2.** NS, not significant.

**Fig. S5. Go enrichment results of Streptomyces-driven host genes in HCC.** The enrichment results can be classified into three major categories: energy and metabolism, immune response, and movement.

**Fig. S6. Multivariate Cox regression analysis was used to evaluate the prognostic significance of the Risk score and clinical parameters.** This result confirms that the risk score provides additional predictive information independent of conventional clinical indicators, and is an independent determinant influencing the prognosis of patients.
